# Virginia

**Published:** 1897-03

**Authors:** E. P. Niles


*Virginia.* Our State Board does not consider dehorning as belonging to the veterinary profession; therefore takes no notice of it when performed by a layman.—*Dr. E. P. Niles.*

